# Disulfiram Oxy-Derivatives Suppress Protein Retrotranslocation across the ER Membrane to the Cytosol and Initiate Paraptosis-like Cell Death

**DOI:** 10.3390/membranes12090845

**Published:** 2022-08-29

**Authors:** Marina Solovieva, Yuri Shatalin, Irina Odinokova, Olga Krestinina, Yulia Baburina, Yana Lomovskaya, Anton Pankratov, Natalia Pankratova, Olga Buneeva, Arthur Kopylov, Alexei Medvedev, Vladimir Akatov

**Affiliations:** 1 Institute of Theoretical and Experimental Biophysics, Russian Academy of Sciences, 142290 Pushchino, Russia; 2 Institute of Mathematical Problems of Biology RAS—The Branch of Keldysh Institute of Applied Mathematics of Russian Academy of Sciences, 142290 Pushchino, Russia; 3 Department of Proteomic Research and Mass Spectrometry, Institute of Biomedical Chemistry, 10 Pogodinskaya Street, 119121 Moscow, Russia

**Keywords:** diethyldithiocarbamate, vitamin B_12b_, DSF oxy-derivatives, ER membrane, ubiquitination, deubiquitinase, paraptosis

## Abstract

Disulfiram (DSF) and its derivatives were here investigated as antineoplastic agents, and their important feature is the ability to influence the UPS. We have recently shown that hydroxocobalamin catalyzes the aerobic oxidation of diethyldithiocarbamate to form disulfiram and its oxy-derivatives (DSFoxy; i.e., sulfones and sulfoxides), which induce cytoplasm vacuolization and paraptosis-like cancer cell death. We used LC-MS/MS and bioinformatics analysis to determine the key points in these processes. DSFoxy was found to induce an increase in the number of ubiquitinated proteins, including oxidized ones, and a decrease in the monomeric ubiquitin. Enhanced ubiquitination was revealed for proteins involved in the response to exogenous stress, regulation of apoptosis, autophagy, DNA damage/repair, transcription and translation, folding and ubiquitination, retrograde transport, the MAPK cascade, and some other functions. The results obtained indicate that DSF oxy-derivatives enhance the oxidation and ubiquitination of many proteins regulating proteostasis (including E3 ligases and deubiquitinases), which leads to inhibition of protein retrotranslocation across the ER membrane into the cytosol and accumulation of misfolded proteins in the ER followed by ER swelling and initiates paraptosis-like cell death. Our results provide new insight into the role of protein ubiquitination/deubiquitination in regulating protein retrotranslocation across the ER membrane into the cytosol and paraptosis-like cell death.

## 1. Introduction

It is known that ubiquitination is a means of regulating the level and functioning of cellular proteins, and the dysfunctions of the ubiquitin–proteasome system (UPS) play an important role in the development of various diseases, including cancer [1]. Studying the ubiquitin–proteasome pathway represents a promising approach to treating human cancer [2]. Dithiocarbamates (DTCs), including disulfiram (DSF) and its derivatives, were here investigated as antineoplastic agents, and their important feature is the ability to influence the UPS. It was found that DSF and other DTCs applied at micromolar concentrations in combination with copper ions enhance the ubiquitination of E3 ligases and inhibit the 26S proteasome, which leads to the accumulation of ubiquitinated proteins (Ub-proteins), the development of ER stress, and, eventually, to apoptotic cell death [1,3,4,5,6,7]. The cytotoxic effect of DSF can be realized through its sulfonic and sulfoxide derivatives, which produce a more damaging effect on numerous cellular enzymes than DSF itself [8,9]. We recently discovered that hydroxocobalamin (vitamin B_12b_) has the ability to catalyze the oxidation of diethyldithiocarbamate (DDC), which was accompanied by the formation of DSF and its sulfones and sulfoxides (DSFoxy) and the synergistic enhancement of the cytotoxic effect of DDC on tumor cells [10]. It was also found that these DSFoxy initiated an unfolded protein response (UPR) in tumor cells, which was accompanied by severe ER stress, ER swelling, and paraptosis-like cell death [11,12]. The initiation of paraptosis-like death is of interest for therapy in cancers characterized by the suppression of apoptosis. In this work, we attempted to evaluate the cause of ER swelling and initiation of paraptosis-like cell death induced by DSFoxy in human carcinoma cells using a mass spectrometric analysis of ubiquitome. Ubiquitome analysis was applied to reveal cellular processes sensitive to the action of DSFoxy, including the transport of proteins across the ER membrane into the cytosol.

## 2. Materials and Methods

### 2.1. Chemicals

DDC was purchased from MPbiomedicals (Irvine, CA, USA). Hydroxycobalamin (vitamin B_12b_) and 5-carboxyfluorescein diacetate N-succinimidyl ester (CFSE) were from Sigma (St. Louis, MO, USA) and Hoechst 33342 from Molecular Probes Inc. (Eugene, OR, USA). Triethylammonium bicarbonate, potassium phosphates, deoxycholic acid sodium salt, urea, and 2-iodoacetoamide were purchased from Sigma (USA); formic acid was from Merck (Darmstadt, Germany); acetonitrile was from Fisher Chemical (UK); and tris-(2-carboxyethyl) phosphine was obtained from Pierce-Thermo Scientific (USA). Trypsin (modified sequencing grade) was obtained from Promega (Madison, WI, USA). N-succinyl-Leu-Leu-Val-Tyr-7-amido-4-methylcoumarin (LLVY-AMC, Sigma St. Louis, MO, USA). Antibodies to ubiquitin (#3933) were from Cell Signaling, and antibodies to GAPDH (#SC-365062) were from Santa Cruz (Santa Cruz, CA, USA). Other chemicals, which were of the highest grade, were from Acros Organics (Waltham, MA, USA).

### 2.2. Cell Culture and Treatment

Human epidermoid carcinoma HEp-2 cells (HeLa-derived human carcinoma cells; ATCC, CCL23) were obtained from the Russian Cell Culture Collection (Institute of Cytology, St. Petersburg, Russia). Cells were grown in DME medium (#5648, Sigma, USA) supplemented with 10% FBS (Gibco, USA), 80 mg/L of gentamycin, and 20 mM sodium bicarbonate at 37 °C in an atmosphere of 5% CO_2_. The cell line was confirmed to be free of mycoplasma infection through regular testing with Hoechst 33342 staining. Cells were seeded in culture dishes (Corning, NY, USA) at a concentration of 4–6 × 10^4^ cells/cm^2^. Freshly prepared solutions of vitamin B_12b_ and filtered DDC were added 24 h after cell seeding.

### 2.3. Immunoblotting

Protein samples (25 µg) were separated by 12.5% SDS–PAGE and transferred to a nitrocellulose membrane. The membrane was blocked with 5% milk (BioRad, Hercules, CA, USA) for 1 h at room temperature and incubated with the primary antibody to ubiquitin (1:500, Cell Signaling, Danvers, MA, USA) at 4 °C overnight. After the incubation with an HRP-conjugated secondary antibody for 1 h at room temperature, the band chemiluminescence was detected by an ECL detection system (ChemiDoc Touch Imaging System, Bio-Rad, USA). GAPDH was used as a loading control.

### 2.4. Analysis of the Chymotrypsin-like Activity of Proteasome

Chymotrypsin-like activity in a crude cell lysate was assayed by measuring the release of the fluorophore 7-amido-4-methylcoumarin from the substrate N-succinyl-Leu-Leu-Val-Tyr-7-amido-4-methylcoumarin (LLVY-AMC, 20 μM). Cell lysates were prepared in homogenizing buffer containing 250 mM sucrose, 5 mM MgCl_2_, 2 mM ATP, 1 mM DTT, 0.025% digitonin, 0.5 mM EDTA, and 50 mM Tris–HCl, pH 7.5, at 4 °C. After the 5 min incubation on ice, samples were centrifugated at 20,000× *g* for 15 min at 4 °C. Proteasome-specific peptidase activity was assayed in assay buffer (50 mM Tris-HCl, pH 7.5, 40 mM KCl, 5 mM MgCl_2_, 0.5 mM ATP, 1 mM DTT, 0.05 mg/mL BSA) [13] on a microplate reader, and changes in fluorescence were calculated against non-treated controls, taking into account the protein concentration in the sample determined by the Bradford method. Samples treated in parallel with the proteasomal inhibitor Bortezomib (1 µM, 4 h) were used as a positive control.

### 2.5. CFSE Staining

Cells were seeded on a Petri dish as described earlier. Twenty-four hours after seeding, control and treated cells were stained with CFSE fluorescent dye (2 µM) within 3 to 4 h after adding DDC+B_12b_. Images were obtained using a TCS SP5 confocal microscope (Leica Microsystems, Mannheim, Germany) and analyzed in Leica Application Suite Advanced Fluorescence 2.1.0 software (Leica Microsystems).

### 2.6. Sample Preparation for Mass Spectrometry

For the assay of protein ubiquitination, three independent experiments were carried out, and the data obtained were combined in a corresponding sample. HEp-2 cells (1.5 × 10^6^) were seeded in culture T25 flasks and treated as described earlier for 1 and 4 h; they were then trypsinized and washed twice with ice-cold PBS. Cell number and viability were estimated through trypan blue exclusion. Cell suspensions were frozen at −70 °C and thawed (three times), then sonicated using a Bandelin 2530 Sonopuls ultrasonic homogenizer (Merck) at ~10% of maximal power for 15 s (4 °C), and proteins were extracted using a chloroform-methanol mixture [14]. The proteins were subjected to trypsinolysis using Vivaspin 500 ultrafiltration devices (10 kDa) (GE Healthcare, Chicago, IL, USA) following [15].

### 2.7. Mass Spectrometry and Liquid Chromatography

High-resolution mass spectrometry analysis was performed as described in [14] using an Orbitrap Fusion (Thermo Scientific, Waltham, MA, USA) with the installed ESI-NSI ion source. The instrument was operated in positive ionization mode with emitter voltage adjusted to 2.2 kV and drying gas temperature at 280 °C. Precursor ions surveyed in a range from 400 m/z to 1200 m/z (maximum integration time was 80 ms) with charge states from z = 2+ to z = 6+ were isolated in the quadrupole mass analyzer within ±1.5 m/z and triggered to fragmentation in the m/z range, with a fixed lower mass (110 m/z) and a dynamic upper mass (depending on the charge state of the fragmented precursor) limited to 2100 m/z. Fragmentation and tandem scanning were performed in an MS3 synchronous precursor ion selection, conditioned by the mass difference between the fragment ions of either ∆M = 114.0429 *u* (corresponding to the ubiquitin tag GG) or ∆M = 383.2281 *u* (corresponding to the ubiquitin tag LRGG), both detected with an asymmetric mass tolerance of −3 ppm/+7 ppm. Only two (N = 2) ions were allowed for synchronous selection in the MS3 mode, provided that the mass difference between ions in pairs was registered.

Liquid chromatography separation was accomplished on an Ultimate 3000 RSLCnano (Thermo Scientific, USA). Samples were loaded onto an enrichment Acclaim µ-Precolumn (0.5 mm × 3 mm, 5 µm) (Thermo Scientific, USA) at a flow rate of 15 µL/min for 3.5 min in 2% acetonitrile, supplied by 0.1% formic acid and 0.03% acetic acid. Analytical separation was carried out at a flow rate 0.3 µL/min using an Acclaim Pepmap^®^ C18 (75 µm × 150 mm, 2 µm) (Thermo Scientific, USA) column with mobile phase A (water with 0.1% formic acid and 0.03% acetic acid) and mobile phase B (acetonitrile with 0.1% formic acid and 0.03% acetic acid) in the following gradient: 2%–37% of mobile phase B for 45 min, followed by column washing in 90% of mobile phase B for 8 min and equilibration of the column under initial gradient conditions (2% of mobile phase B) for 15 min before starting the next run.

Data files obtained after LC-MS/MS analysis were converted into a peak list format and used for protein identification. Proteins were identified against a concatenated target/decoy database of human proteins (UniProt database revision April 2019). The decoy sequences were created by reversing the target sequences in SearchGUI. The identification settings were as follows: trypsin (specific), with a maximum of three missed cleavages, with ±5.0 ppm tolerance at the MS1 level and ±0.03 Da tolerance for MS2 tolerances. The following variable modifications were set: carbamidomethylation of C (+57.021464 Da), oxidation of M (+15.994915 Da), ubiquitination of K as a GG tag (+114.042927 Da), and long ubiquitination tag of K (+383.228102 Da). Variable modifications were refined after the search procedure. Peptides and proteins were inferred from the spectrum identification results using PeptideShaker version 1.16 (Compomics, Gent, Belgium). Peptide spectrum matches (PSMs), peptides, and proteins were validated at a 1.0% false discovery rate (FDR) estimated using the decoy hit distribution. Proteins were classified in terms of Gene Ontology (GO) annotations using the STRAP software (version 1.5.0.0) [16].

### 2.8. Bioinformatics Analysis of Ubiquitome

Functional analysis of ubiquitome was performed in PANTHER version 16.0, released 1 December 2020 [17]. The analysis type used was the PANTHER Overrepresentation Test (released 20210224). The lists analyzed were D4h, DB1h, and DB4h versus the reference list (control). Annotation datasets were the PANTHER GO-slim Molecular Function (MF), Biological Process (BP), Cellular Component (CC), Protein Class (PC), and PANTHER pathways. The test type used was the binomial test without Bonferroni correction. First, the ontologies were obtained for which statistically significant deviations from the control for fold enrichments were recorded. The fold enrichment of each ontology was equal to the relative number of proteins in the analyzed list divided by the relative number of proteins in the reference list. Then, the results obtained were subjected to additional manual selection: ontologies for which a positive regulation by a factor of two or more was found in at least one of the samples. The gene ontologies of identified ubiquitinated proteins were divided into four subcategories: GO-slim BP, MF, CC, and PC.

Individual Ub-proteins were also analyzed and grouped according to the UniProtKB (release January 2021) keywords describing the main biological processes/molecular functions/cellular components (response to exogenous stress, including response to heavy metal ions, hydrogen peroxide, ethanol, cocaine and other drugs, heat, osmotic stress, and other types of stress; proteins involved in mitochondria and energy metabolism; apoptosis and DNA damage/repair; cell cycle regulation; autophagy and necroptosis; ER; Golgi; folding and ubiquitination (since many proteins involved in the UPR, UPS, ERAD, Ubl conjugation, and protein ubiquitination pathways are implicated in several of these processes, we combined these proteins into one group); transcription; translation; cytoskeleton; Ca^2+^-related proteins; phosphatidylinositol (PI)-related proteins; lipid metabolism; and proteins related to the transport of Na^+^, K^+^, and Cl^−^ ions). If a protein performed several functions that we selected for research or participated in several processes of interest, it was assigned to several groups. Oxidized proteins of each sample were also grouped and analyzed.

All ubiquitome and UniProtKB groups of Ub-proteins were searched against the STRING database, version 11.5 [18], for protein–protein interactions.

### 2.9. Statistical Analysis

Each experiment was performed at least three times. All the values represent the means ± s.e.m. The statistical significance of the results was analyzed using the Mann–Whitney U test.

## 3. Results

### 3.1. Effect of Exogenous DSFoxy on Protein Ubiquitination

**Figure 1:**
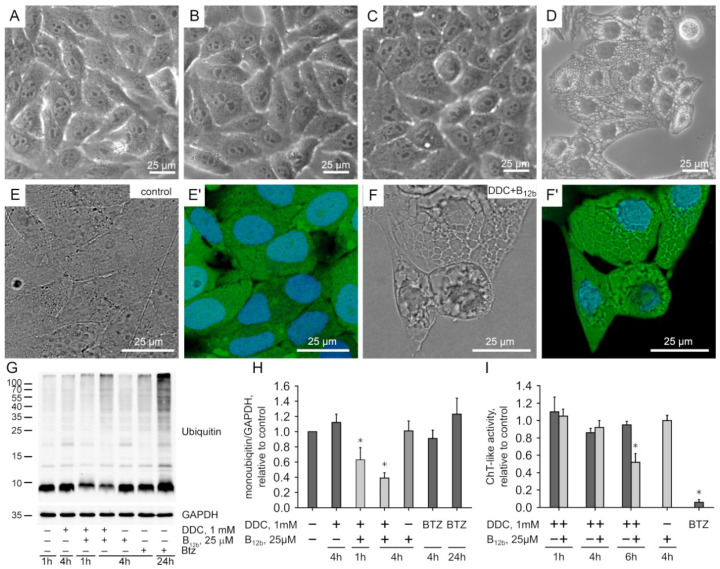
Oxidation of DDC catalyzed by B_12b_ induces vacuolization and paraptosis-like death of HEp-2 cells. (**A**–**D**) Phase-contrast images of cytoplasmic vacuoles in HEp-2 cells. (**A**) Untreated cells. (**B**) Cells treated with DDC (4 h). (**C**,**D**) Cells treated with DDC+B_12b_ for 1 h and 4 h. (**E**,**F**) Confirmation of protein synthesis in the vacuoles of HEp-2 cells during the initiation of their death through staining with 2 µM CFSE from 3 to 4 h of incubation with DDC+B_12b_. (**E**,**E’**) Control. (**F**,**F’**) Cells treated with DDC+B_12b_ for 4 h. (**G**,**H**) Immunoblot analysis of protein polyubiquitination and monoubiquitin levels in HEp-2 cells. (**I**) Estimation of 26S proteasome activity of HEp-2 cells during the initiation of their death using 1 mM DDC combined with 25 μM B_12b_. The data are the means ± s.e.m. of three separate experiments. *, Significant differences between the samples and the control, *p* < 0.05.

The incubation of HEp-2 cells for 4 h with the combination of DDC (1 mM) and B_12b_ (25 µM), but not with B_12b_ or DDC alone, led to irreversible vacuolization (Figure 1A–D) and initiated cell death [11]. We applied cell staining during vacuolization with CFSE, which, after the hydrolysis by esterase, binds to amino groups and, thus, stains protein molecules. We found that the vacuoles also contained a dye, which confirms our previous data [11] on the ongoing protein synthesis in the ER during vacuolization (Figure 1F). Using immunoblotting, we found that the incubation of HEp-2 cells with 1 mM DDC taken alone and vitamin B_12b_ did not induce changes in either the protein polyubiquitination or monomeric ubiquitin levels (Figure 1G,H). Combination of DDC with B_12b_ caused a pronounced decrease in the amount of monomeric ubiquitin by 1 h, which became more significant by 4 h, and increased the number of polyubiquitinated proteins by 4 h of incubation (Figure 1G,H, Supplementary Figure S1A).

An increase in the number of polyubiquitinated proteins can result from a fall in the activity of the proteasomal system of protein degradation. We detected only a slight inhibition of the chymotrypsin-like activity of cell lysates during 1–4-h of incubation with DDC+B_12b_. A significant decrease in proteasomal activity was observed by 6 h, just before the onset of cell death (Figure 1I, Supplementary Figure S1B). Bortezomib inhibited the chymotrypsin-like activity of the proteasome and also caused a marked accumulation of polyubiquitinated proteins by 4 h, but small vacuoles appeared in a part of the cell population only at 24 h of incubation (Figure 1; Supplementary Figure S1C,D). Based on these results, we used the following samples for the study of the ubiquitome: (1) untreated human carcinoma HEp-2 cells—control; (2) HEp-2 cells collected after 4 h incubation with 1 mM DDC, which did not cause cell death—D4h sample; (3) HEp-2 cells after 1 h incubation with 1 mM DDC and 25 µM B_12b_, which caused neither vacuolization nor a cytotoxic effect—DB1h sample; and (4) HEp-2 cells after 4 h incubation with 1 mM DDC and 25 µM B_12b_, which irreversibly induced vacuolization and cell death [11]—DB4h sample.

### 3.2. Functional Analysis of Ubiquitome in PANTHER

**Figure 2:**
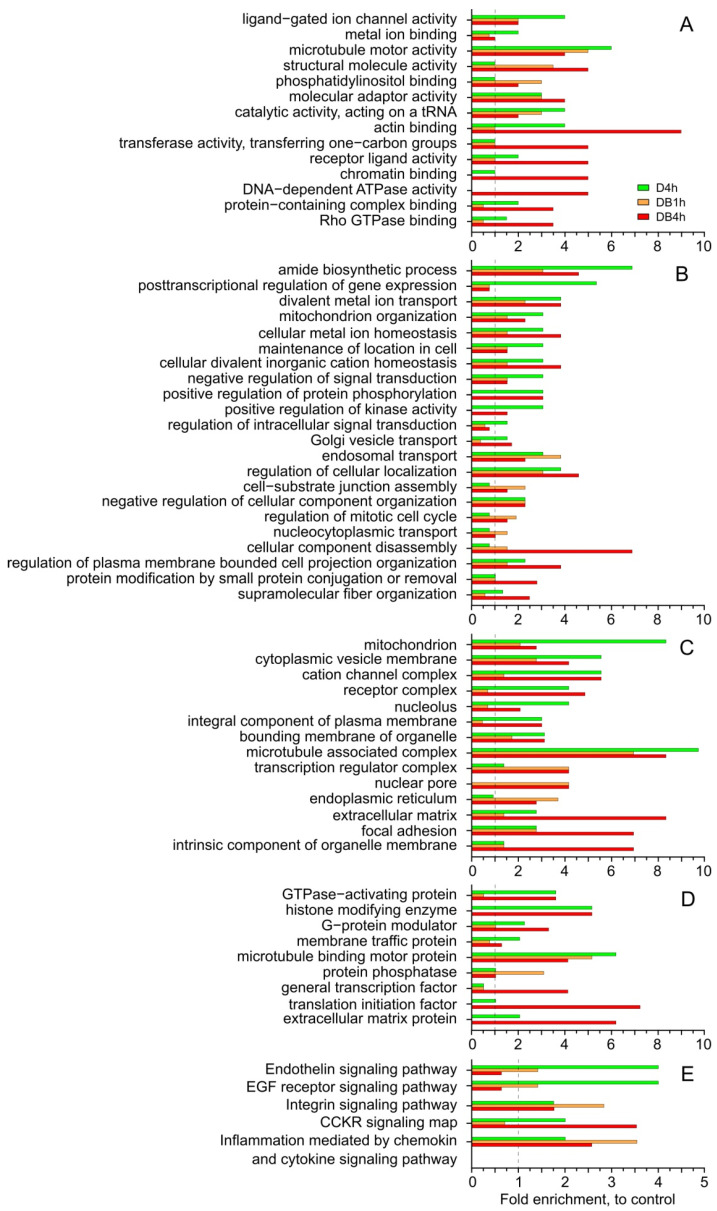
Enrichment analysis of PANTHER GO-slim annotation in experimental samples relative to untreated control HEp-2 cells. (**A**) Molecular functions (MFs). (**B**) Biological processes (BPs). (**C**) Cellular component (CCs). (**D**) Protein class (PCs). (**E**) Signaling pathways. The *y*-axis shows the groups with significantly enriched values (*p* < 0.05).

Using mass spectrometry, we identified 388 ubiquitinated sites and 321 ubiquitinated proteins (Ub-proteins) in untreated control cells, 429 ubiquitinated sites and 315 Ub-proteins in the D4h sample, 315 sites and 227 Ub-proteins in DB1h, and 612 sites and 480 Ub-proteins in DB4h (Supplementary Table S1). The number of ubiquitinated sites in proteins varied from one to three in the control, and there were four in the other samples. Ub-proteins in the samples were characterized with the help of the Gene Ontology (PANTHER GO-slim [17]) functional classification into five independent categories: molecular functions (MFs), biological processes (BPs), cellular components (CCs), protein class (PC), and PANTHER pathways (Figure 2, Supplementary Table S1).

The number of Ub-proteins in the DB4h sample relative to control values was higher than that in the D4h sample for the following MFs (Figure 2A): structural molecule activity, transferase activity, transferring one-carbon group, actin binding, receptor ligand activity, Rho GTPase binding, protein-containing complex binding, and chromatin binding. Proteins involved in DNA-dependent ATPase activities were ubiquitinated only in the DB4h sample. In this sample, increases in the numbers of Ub-proteins involved in BPs, such as cellular component disassembly, protein modification through small protein conjugation or removal, supramolecular fiber organization, and regulation of plasma membrane-bounded cell projection organization, were more significant than in D4h (Figure 2B). The numbers of Ub-proteins associated with some CCs (endoplasmic reticulum, nuclear pore, transcription regulator complex, intrinsic component of organelle membrane, focal adhesion, extracellular matrix) in the DB4h sample were greater than in D4h (Figure 2C). Analysis of the PC category revealed excesses for the increased numbers of Ub-proteins in the DB4h sample compared to in D4h for the translation initiation factor, general transcription factor, and the extracellular matrix protein (Figure 2D). Compared to the control, the numbers of Ub-proteins in the DB4h sample were higher than in DB1h for almost all the PC categories studied; however, for microtubule-binding motor protein and protein phosphatase, they were lower (Figure 2D).

The PANTHER GO-slim analysis revealed activation of the inflammation signaling pathways mediated by chemokine and cytokine signaling, as well as integrin signaling pathways, in all experimental samples. In DB4h, the number of Ub-proteins of the CCKR signaling map pathway was significantly increased (Supplementary Table S1, Figure 2E).

### 3.3. Individual Analysis of Ub-Proteins in Groups Formed According to the Classification of UniProtKB

**Figure 3:**
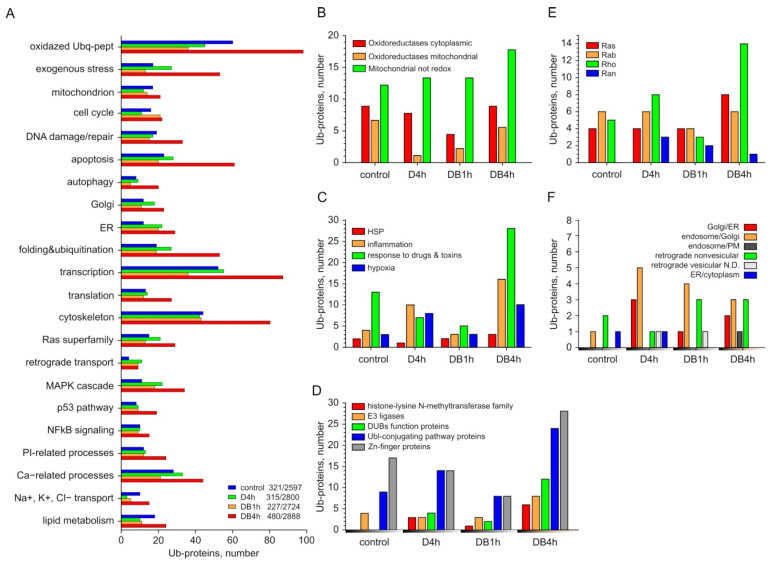
The numbers of Ub-proteins found in DB4h, DB1h, D4h, and control samples in different UniProtKB groups. (**A**) General analysis by groups. The number of all Ub-proteins versus the total number of proteins detected in the samples is indicated. (**B**) The increase in the numbers of cytoplasmic and mitochondrial Ub-oxidoreductases and mitochondrial non-redox Ub-proteins. (**C**) The numbers of Ub-proteins of some intracellular systems that respond to exogenous stress; (**D**) histone-lysine N-methyltransferase family, E3 ligases, DUBs’ function proteins, Ubl-conjugating pathway proteins, and zinc finger proteins. (**E**) Ras superfamily and (**F**) retrograde transport.

The standard analysis of PANTHER GO-Slim did not reflect the effects that we found experimentally; e.g., the inhibition of the apoptotic pathway of cell death by DSFoxy [11]. Therefore, we carried out an additional analysis of Ub-proteins grouped according to UniProtKB keywords and estimated protein oxidation in all samples. As shown in Figure 3A, the numbers of Ub-proteins significantly increased in most selected groups of the DB4h sample and they were nearly equal or decreased in most groups of D4h and DB1h samples compared to the control values. The numbers of oxidized Ub-proteins substantially (by 60%) increased in the DB4h sample and decreased in the DB1h and D4h samples compared to the control (Figure 3A, Supplementary Table S2(1)). The oxidation of cellular proteins may have been related to the activity of oxidoreductases, which can also mediate the formation of disulfide bonds during protein folding [19]. A noticeable decrease in the number of ubiquitinated mitochondrial oxidoreductases was detected in all experimental samples, and the number of ubiquitinated cytoplasmic oxidoreductases in the D4h and DB4h samples compared to the control remained unchanged. At the same time, an increase in the number of non-redox Ub-proteins in mitochondria (Figure 3B, Supplementary Table S2(2)) was revealed in the DB4h sample, with no significant change in the total number of Ub-proteins of a mitochondrion relative to the control. The number of Ub-proteins associated with energy homeostasis decreased in DB1h and slightly increased in the DB4h sample (Supplementary Table S2(3)).

Both the sulfones and sulfoxides of DSF and DDC themselves are xenobiotics for cells. We found that the number of Ub-proteins with the function we designated as “response to exogenous stress” (see Section 2) was slightly decreased in the DB1h sample as compared to the control, and in the DB4h sample, it was many times greater than in all other samples (Figure 3A,C, Supplementary Table S2(4)).

Enhanced ubiquitination of some key proteins related to the DNA damage checkpoint was found in the DB1h sample. In the DB4h sample, a lot of histone-lysine N-methyltransferase family proteins involved in the response to DNA damage, transcription, and histone and chromatin binding were detected (Figure 3D, Supplementary Table S2(5)). The ubiquitination and oxidation of apoptosis regulators increased several times, and the increase in the number of proteins related to DNA damage/repair was also noticeable (Figure 3A, Supplementary Table S2(5d)), in contrast to other samples. In the DB4h sample, the number of Ub-proteins associated with the regulation of autophagy and macroautophagy also substantially increased, in contrast to other samples (Figure 3A). In the D4h sample, we found enhanced ubiquitination of the RPTOR protein, which regulates cell survival, macroautophagy, and the G1/S transition in the cell cycle. In addition, the ubiquitination of proteins involved in the regulation of necroptosis was recorded in DB1h and DB4h samples (Supplementary Table S2(6)).

In the DB4h sample, the numbers of Ub-proteins for the ER and Golgi were two times greater than in the control and exceeded those found in DB1h and D4h (Figure 3A, Supplementary Table S2(7)). One of the earliest cellular responses to ER stress is the inhibition of protein synthesis in order to reduce the load on the ER. A significant increase in the number of Ub-proteins associated with the regulation of transcription and translation, including Zn-finger proteins and members of the eIF-4 family, etc. (Figure 3A,D, Supplementary Table S2(8)), was found only in the DB4h sample. The number of Ub-proteins in the “folding and ubiquitination” group in this sample was more than 2–2.8 times higher than that in the other samples (Figure 3A, Supplementary Table S2(9)). In the DB4h sample, we found an increase in the number of Ub-proteins of the UBL-conjugation pathway, including E3 ligases, ubiquitin C-terminal hydrolases, and deubiquitinating proteins (Figure 3D). Three out of eight E3 ligases were oxidized. The number of oxidized proteins (fourteen) in the “folding and ubiquitination” group in the DB4h sample was many times greater than in the other samples (six in the control sample, two in DB1h, and one in D4h). In experimental samples, we identified polyubiquitin B and Ub-proteins from the cullin family, which function as a scaffold for E3 ubiquitin-protein ligase complexes, and other proteins that take part in these processes (DnaJ, F-box, etc.).

We only detected a significant increase in the number of Ub-proteins associated with the Ras superfamily, including the Rho family regulating the development of organelles and the dynamics of the cytoskeleton, in the DB4h sample (Figure 3E, Supplementary Table S2(10)), which correlates well with the increase in the ubiquitination of cytoskeleton-related proteins (Figure 3A, Supplementary Table S2(11)) and the significant decrease in the level of polymerized actin that we found in MCF-7 cells by 4–6 h of treatment with DSFoxy [12]. The Ub-proteins of the Ran family were found in all experimental samples. The key regulators of the vesicular membrane traffic RAB30, RAB5C, and oxidized RAB26 were found only in DB samples. The ubiquitination of proteins participating in the regulation of non-vesicular and endosome/PM retrograde transport was increased in the DB4h compared to the D4h sample (Figure 3F, Supplementary Table S2(12)). About half of the Ub-proteins involved in retrograde transport in the DB1h and DB4h samples were oxidized (four out of nine and three out of nine, respectively), which was distinct from the D4h sample. Of interest in Figure 3E is the absence of Ub-proteins transferring damaged and unfolded proteins out of the ER into the cytoplasm in samples DB1h and DB4h, in contrast to the control and D4h. We found no significant difference in the ubiquitination of proteins involved in protein transport between experimental and control samples (Supplementary Table S2(12)). The numbers of Ub-proteins associated with the MAPK cascade; phosphatidylinositol (PI) metabolism and regulation of IP3 kinase (PI3K); NF-kB; p53 signaling; Ca^2+^-dependent processes; the transport of Na^+^, K^+^, and Cl^−^ ions; and lipid metabolism in the DB4h sample were increased compared with the other samples (Figure 3A, Supplementary Tables S2(13)–(17)). In D4h and DB1h, the numbers of Ub-proteins in some of the abovementioned groups were even significantly less than in the control.

### 3.4. Protein Interaction Network Analysis

Using the STRING database, we attempted to determine whether the ubiquitinated proteins we found were associated with each other. An analysis of the complete ubiquitome made it possible to identify several clusters in which most proteins showed close functional interactions with each other and with the proteins of other clusters (Supplementary Figure S2). These included the clusters of actin- and ubiquitin-associated proteins and the “nucleus-DNA” cluster and its associated clusters CDC5L and SYNE1 (the proteins of which participate in nucleocytoplasmic interactions in particular) (Supplementary Figure S2A–G). At the same time, a great number of Ub-proteins showed no links with other proteins.

**Figure 4:**
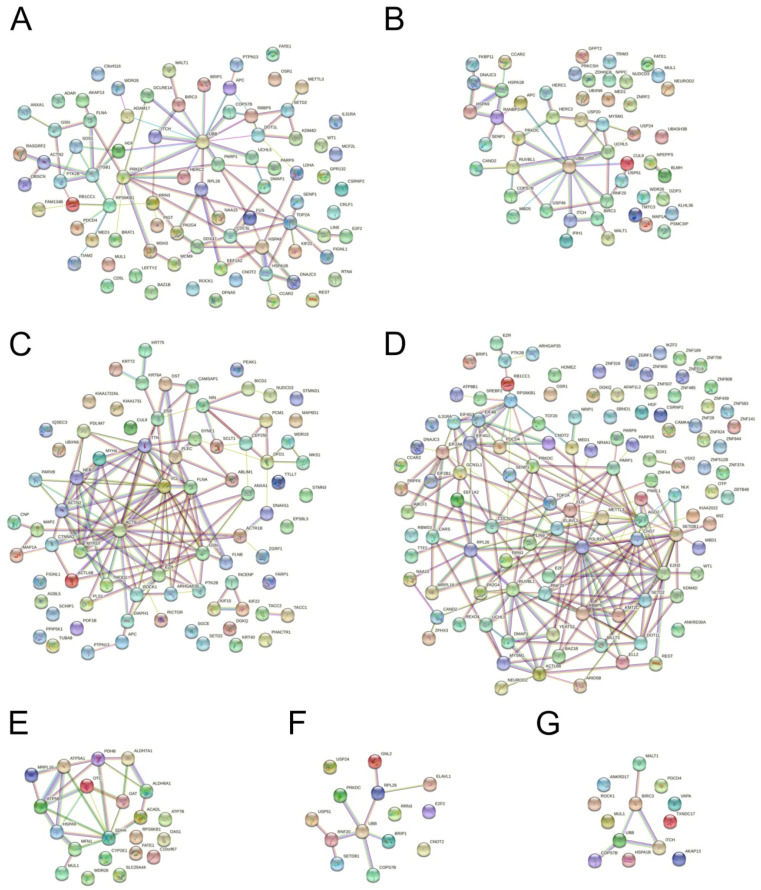
PPI analysis using STRING database: a significant enrichment in groups of Ub-proteins selected by UniProtKB keywords. (**A**) Apoptosis and DNA damage/repair, *p*=0.0164. (**B**) Folding and ubiquitination, *p* = 1.6 × 10^−9^. (**C**) Cytoskeleton, *p* < 1.0 × 10^−16^. (**D**) Transcription and translation, *p* < 1.0 × 10^−16^. (**E**) Mitochondrial proteins, *p* =3.1 × 10^−11^. (**F**) p53, *p* = 0.0126; and (**G**) NfkB, *p* = 0.0394. For each group, the PPI enrichment *p*-value is indicated.

The analysis using the STRING database showed that the ubiquitinated proteins in most of the UniProtKB groups we identified were functionally associated (Figure 4). Thus, most of the proteins in the apoptosis and DNA damage/repair groups showed functional interactions with UBB, PRKDC, and TOP2A (Figure 4A). Multiple PPIs were identified in the following groups: folding and ubiquitination, cytoskeleton, transcription and translation, NfkB, and p53 (Figure 4B–G). Zn-finger proteins with associations with the remaining proteins of transcription that were not revealed in the STRING database could, according to UniProt, also play a role in this process. More than half of the proteins in the group of mitochondrial proteins had multiple associations: among others, with HSPA9, the subunits of ATP synthase, MFN1, etc. (Figure 4E). Despite the heterogeneity of the group that combined the proteins involved in response to different types of stress, functional interactions here were significantly increased (Supplementary Figure S3A, *p* < 0.05). In the group of Ub-proteins that regulated the metabolism and transport of lipids, significant PPI enrichment was found, despite the fact that most of these proteins were not associated with each other; the same was true for the Ub-proteins of the RAS family, PI- and Ca-metabolism, and autophagy (Supplementary Figure S3B–F). According to the STRING database, no increase in PPI was exhibited in the groups for MAPK signaling and regulation of K^+^, Na^+^, and Cl^−^ transport (Supplementary Figure S3G,H, *p* > 0.05).

## 4. Discussion

As can be seen from the analysis performed, there was no increase in the total number of Ub-proteins in the D4h sample, where cell death was not observed [11]. A change in the protein distribution in the selected groups occurred: increases in the numbers of Ub-proteins in the ER and Golgi, those related to the regulation of retrograde transport, and those related to the MAPK cascade, as well as those of some MFs, BPs, CCs, and PCs. These changes may reflect the adaptive response of cells to the cytostatic effect of DDC, as ubiquitination may play a non-proteolytic regulatory role in DNA repair, translation, inflammatory response, and other physiological processes [20,21,22]. According to the STRING database, in this sample, as well as in the control, post-translational modification (PTM) of proteins with the participation of ubiquitin took place (GO:0043687 post-translational protein modification, *p* < 0.05).

As was shown earlier, the DB1h sample exhibited non-lethal changes in the cell that preceded irreversible damage induced by DSFoxy in the DB4h sample [11]. The total number of identified Ub-proteins in the DB1h sample was reduced compared to the control. The analysis of UniProtKB groups in the DB1h sample revealed increased numbers of various Ub-proteins related to cell cycle regulation, DNA damage/repair, and the Ubl conjugation pathway (Supplementary Table S2). As discussed, ubiquitination is a means for the physiological regulation of these functions. However, the ubiquitination of an important protein such as MDC1 leads, according to UniProt, to its further degradation by the proteasome, and the activation of this process in DB1h may indicate the start of inhibition of the DNA damage/repair system. The increased representation of the ubiquitinated 60S ribosomal protein L26 (RPL26) in the DB1h and DB4h samples (Supplementary Table S2(5b,d)) may have indicated the response to DNA damage and ER stress, similar to UFMylation by UFL1 [23]. According to the STRING database, no ubiquitin-mediated PTM was found among the biological processes of the DB1h sample, which may indicate the role of ubiquitination in the breakdown of proteins of this sample.

The DB4h sample, obtained from cells during their preparation for death, was characterized by noticeable differences in the number of ubiquitinated proteins. The increased total number of Ub-proteins in this sample relative to the control was in good agreement with the WB and BP analysis (Figure 1 and Figure 2B). The damaging effect of DSFoxy may have been evidenced by the increased number of ubiquitinated proteins that respond to exogenous stress and metabolize xenobiotics; particularly, cytochrome P450 localized in the ER. These data are consistent with the inactivation of CYP2E1 by DSF derivatives and its ubiquitination and further proteasomal degradation [24,25]. They may indicate damage to the cellular detox system in the DB4h sample. Pathway analysis revealed that inflammation was mediated by chemokine and cytokine signaling pathways, as well as integrin signaling pathways, and it was more activated compared to other experimental samples’ CCKR signaling maps, which was in good agreement with the analysis of individual Ub-proteins. As can be seen from the results shown in Figure 3, in the DB4h sample, the numbers of Ub-proteins in almost all selected groups were greater than in DB1h, with a few exceptions (Figure 2 and Figure 3), and ubiquitination was found in the proteins of the most important cell organelles. As indicated above, ubiquitination, along with the regulation of various cellular functions, is also an important step in the degradation of damaged proteins [14,20,21,22]. The oxidation in the DB4h sample of Ub-proteins such as PA2G4, ROCK1, FLNA, TTN (which contains >20 disulfide bonds and was not oxidized in other experimental samples), CENPL and CEP250, PHKA2, DST, proteins of the Golgin subfamily A, MAP1A and MAP2, TMTC3, RyR1, MCM5 and MCM7, TTLL7, FATE1, KIF22 and KIF15, and those that regulate vesicular transport and other important functions indicated damage to the nucleus and DNA, cytoskeleton [26], mitochondria, ER, Golgi apparatus, and UPS and may explain the observed fatal effects of DSFoxy. DSF derivatives can both initiate and inhibit apoptotic cell death [3,4,5,6,7,27,28] and autophagy [29]. As we have found previously [11], the death of HEp-2 cells induced by DSFoxy does not occur through autophagy and apoptosis. The individual UniProtKB analysis showed that highly reactive DSFoxy significantly increase the ubiquitination of proteins associated with the regulation of apoptosis, necroptosis, and autophagy. The ubiquitination-induced PTM of the various proteins regulating these processes, as well as DNA repair and cell cycle progression, as identified in our work, can precede their proteasomal degradation. This is known, in particular, for the F-box 5 protein, as well as cysteine endopeptidases BIRC3, PDCD4, and MUL1 [20,22]. However, according to the STRING database, no ubiquitin-mediated PTM was found among the biological processes occurring in this sample, indicating the degradative nature of protein ubiquitination. Similarly to ALDH, protein kinase C, P-glycoprotein, DNA methyltransferases, and caspase 3 [8,9,29,30], the sulfhydryl groups of the Ub-proteins mentioned above can be attacked and changed/oxidized by DSFoxy, which just leads to the blockage of apoptosis.

One of the early and important events in the damaging action of DSFoxy is the stress in the ER caused by the accumulation of protein molecules in its cisternae (Figure 1). The absence of a drop in the total number of proteins in the samples (Figure 3), the presence of a protein-binding CFSE dye in vacuoles (Figure 1), and the increased quantity of ubiquitinated proteins regulating transcription and translation in DB4h (Figure 2B,D and Figure 3) suggest that protein synthesis continues, possibly due to attempts to replenish unfolded proteins, which in turn causes further ER overload [31]. It is known that newly synthesized proteins and the master regulators of their folding in the ER are very sensitive to the action of SH-modifying agents, which include sulfoxide derivatives of DSF. UPR regulators (e.g., PERK) are known to be inhibited by DTC sulfoxides, ultimately leading to severe ER stress [32]. This explains the protective effect of cycloheximide and 4-PBA, observed at early stages of death initiation in HEp-2 cells [11]. Folding of proteins in the DB4h sample is substantially impaired, as evidenced by the accumulation of ubiquitinated and oxidized UPR regulators (Supplementary Table S2(9)) [11]. Thus, we found the ubiquitination of ZDHHC6 palmitoylating calnexin, which, according to UniProt, is required for its association with the ribosome-translocon complex and for efficient folding of glycosylated proteins. Regulators of folding—PTM and ER stress response PRKCSH (glucosidase 2 subunit beta), FKBP11 (peptidyl-prolyl cis-trans isomerase), and DNAJC3 (DnaJ homolog subfamily C member 3)—were also ubiquitinated, and TMTC3 (protein O-mannosyl-transferase) was oxidized.

For normal cell functioning, misfolded proteins and immature Golgi proteins must be returned to the ER, and unfolded or damaged proteins must be delivered from the ER to the cytosol, which can be achieved in several ways [33]. PERK, IRE1α, and ATF6 activate the processes that stimulate the retrograde transport of unfolded proteins out of the ER for ubiquitination and further proteasomal degradation [34]. The circulation of chaperones (e.g., BiP), ER export factors, and vesicular transporters is also carried out via ubiquitination-regulated retrograde transport [35,36]. Increased numbers of ubiquitinated and oxidized Rab proteins known to regulate vesicular transport, as well as those of other members of this superfamily and the proteins interacting with them, were found in DB4h (Figure 3C). The ubiquitination of proteins involved in the retrograde transport found in the D4h, DB1h, and DB4h samples may indicate the activation of these processes to counteract the accumulation of misfolded or damaged proteins. Oxidation of these proteins in DB1h and DB4h samples may indicate its impairment and the suppression of this process. Of particular interest for understanding the mechanism underlying the initiation and progression of ER stress were our data about the ubiquitination of proteins responsible for the retrograde transport of proteins, first of all out of the ER into the cytoplasm. Since protein synthesis continues, the accumulation of proteins in the ER is possible if the system for the control and transport of damaged or misfolded proteins to the retrotranslocon is disturbed. The absence of ubiquitinated proteins involved in the ER/cytoplasm retrograde transport in the DB4h and DB1h samples, in contrast to the control and D4h samples, may indicate that these proteins do not exit from the ER into the cytosol. In particular, this may be the case with the protein OS-9, which binds to damaged or misfolded proteins after their quality checking in the ER and takes part in their transfer to the translocon. Damage to these proteins prevents the exit of injured or misfolded proteins out of the ER into the cytoplasm and promotes, in the presence of actively functioning translocon, the accumulation of these proteins in the ER, thereby initiating vacuolization and severe ER stress. The increase in the IRE1alfa and PERK contents that we found earlier in DB1h [11], along with the further threefold decrease in DB4h, may also indicate inhibition of the retrotranslocation, since IRE1alfa is an activator of XPB-1 protein that binds to promoters of several genes involved in retrograde transport of misfolded proteins from the ER to the cytosol and in ER-induced protein degradation [37].

Another important member of cell proteostasis regulation is the ubiquitin–proteasome system (UPS). Ubiquitination and the delivery of damaged proteins to proteasomes (which, as shown in Figure 1F, retained high activity in the DB4h sample) are carried out with the participation of E1, E2, and E3 ubiquitin-modifying enzymes. It has been reported previously that DSF impairs UPS functioning; namely, it causes the degradation of UBE1 protein [31] and inhibits the self-ubiquitination and activity of RNF115/BCA2 E3 ligase through the binding and ejection of zinc [1,38,39]. Our results showing significant increases in the numbers of ubiquitinated E3 ligases (most of which contained cysteine-rich Zn-fingers) and ubiquitin C-terminal hydrolases in the DB4h sample (Figure 3F) also indicate the ability of DSFoxy to impair UPS functioning. In addition to the transfer of the ubiquitin molecule to the protein to be cleaved and the translocation of misfolded proteins from the ER to the proteasome [40], E3 ligases can participate in the repair of damaged proteins and membrane trafficking. Examples of these enzymes may include HERC2 and HERC1, the ligases of the HECT type, which contain an active Cys residue [20]. These enzymes were oxidized in the DB4h sample, but HERC2 and other ligases in the D4h sample were not oxidized. According to the UniProt database, the E3 ligases we detected in the DB4h sample are activated without ubiquitination. Theoretically, E3 ligase ITCH in DB4h can be autopolyubiquitinated, which would not lead to protein degradation [41]. However, we found that this HECT-type E3 ligase was oxidized in DB4h, so its ubiquitination is an indicator of impairment. Thus, the ubiquitination of E3 ligases may indicate both its load with ubiquitin chains before the reaction with a damaged protein molecule and the disturbance in UPS activity due to self- or trans-E3-ubiquitination prior to degradation [20]. For example, the polyubiquitination of E3 SUMO-protein ligase RanBP2 containing Zn-fingers and disulfide bonds leads to proteasomal degradation.

The regulators of the final stage of protein transfer to the proteasome are deubiquitinases (DUBs), including ubiquitin C-terminal hydrolases associated with the 26S proteasome. These enzymes often also contain a sulfhydryl group in the active center [35], the damage to which by DSFoxy probably explains the increased ubiquitination of these enzymes in DB samples compared with the control and D4h samples. UCHL5, associated with the 19S regulatory subunit of the 26S proteasome, was ubiquitinated and oxidized in the DB4h sample. MYSM1 (thiol-dependent DUB) and many proteins with deubiquitinase activity were also ubiquitinated in DB4h. This suppression of DUB activity may have been the reason for the decrease in the levels of monoubiquitin in the DB1h and DB4h samples (Figure 1). Some DUBs function as regulators of retrotranslocation (for example, of the Nrf1 and Hrd1 complex) and E3 enzyme stability [19,42,43,44,45,46]. Blocking the deubiquitinase activity can also be one of the main reasons for the shutdown of the “molecular conveyor” of proteins from the ER to the proteasome and the accumulation of misfolded proteins in the ER, ultimately leading to catastrophic vacuolization.

In experimental samples, in contrast to the control, we found more ubiquitinated proteins of the Ras family; in particular, the Ran subfamily, which is essential for the translocation of RNA and proteins through the nuclear pore complex (Figure 3D). It is known that the nucleus also contains a proteasome system for quality control and degradation of proteins, both nuclear and cytoplasmic, and the proteasome can be associated with nuclear pores [47,48,49]. This system could also be inhibited by DSFoxy. Thus, the nuclear pore complex protein Nup98-Nup96 was oxidized in the DB1h sample. In the DB4h sample, ubiquitinated RANBP2, a component of the nuclear pore, was found (see above). The proteins of the Ras superfamily transmit a signal from extracellular stimuli to MEK, ERK, and other targets, including NF-kB and p53. DTCs inhibit NF-kB activation, translocation, and DNA-binding activity [50,51,52], as well as p53 glutathionylation and degradation [53]. The role of p53 in paraptosis is currently under extensive investigation since it has been shown that paraptotic cell death can be effectively induced in several p53-defective cancer cell lines [54,55,56]. The proteins mentioned above can bind p53 (BRE1A) and regulate p53/TP53 expression (ELAVL1), p53 signal transmission by mediators (BARD1), and the signaling of I-kappaB kinase/NF-kappaB (BIRC3, MUL1, MALT1, and PDCD4). One aspect of MAPK signaling is the PI3K-AKT pathway, which is activated by many types of stress; its main function is to inhibit apoptosis and promote proliferation. A large number of Ub-proteins associated with MAPK signaling, as well as with p53 and phosphatidylinositol (PI) metabolism, were found in the DB4h sample. The activation of the MAPK cascade is a characteristic of paraptosis [54,57] and the CCKR signaling map.

Protein–protein interactions (PPIs) in most groups that we identified were significantly enhanced, which underlines the adequacy of the approach for the grouping of proteins. The STRING analysis of PPI sin the DB4h sample made it possible to mark BIRC3, ITCH, PTK2B, and HSPA1A, which were included in all main groups (response to stress, regulation of apoptosis, folding and ubiquitination, participation in the MAPK-cascade/p53/NF-kappaB pathway) and regulate the reorganization of the cytoskeleton, transcription, and many other processes. BIRC3 and ITCH are functionally directly associated with UBB and PTK2B through ITGB1 and HGF, while HSPA1A is associated with them through TOP2A; and PARP1. However, the available data were insufficient to determine the role of these proteins in the initiation of paraptosis.

## 5. Conclusions

**Figure 5:**
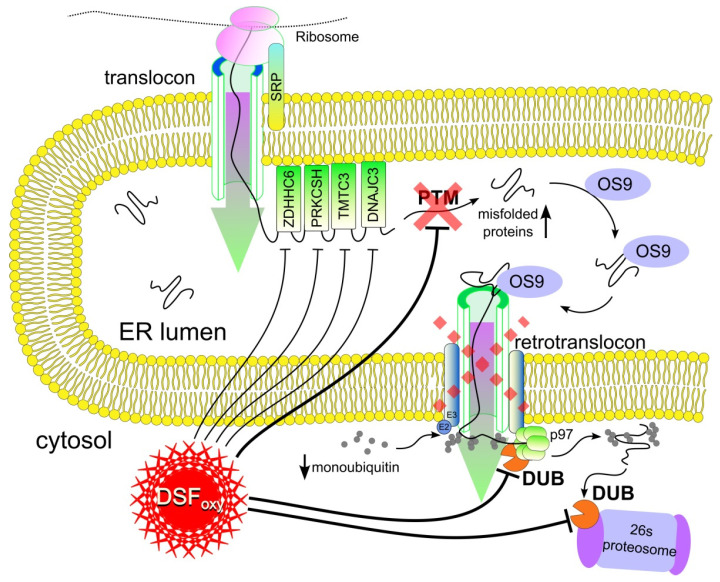
Scheme representing the proposed mechanism of the cytotoxic action of DSF oxy-derivatives, the key point of which is the suppression of the UPS mediated by DUB damage, followed by inhibition of retrotranslocation of proteins across the ER membrane into the cytosol, the accumulation of misfolded proteins in the ER, and the initiation of paraptosis-like cell death.

Our results make it possible to propose a mechanism by which DSF oxy-derivatives formed in the reaction of DDC with B_12b_ produce a cytotoxic effect. These DSFoxy cause the oxidation and impairment of a variety of proteins, including those involved in the regulation of apoptosis and autophagy, and the inhibition of these means of cell death. The key point in the mechanism of DSFoxy-mediated cell death can be assumed to be the damage to deubiquitinases associated with the ubiquitin–proteasome system, which leads to suppression of the UPS followed by the inhibition of the retranslocation of damaged and misfolded proteins across the ER membrane into the cytosol. This suppression of protein retranslocation across the ER membrane, in combination with the ongoing protein biosynthesis, can lead to the accumulation of defective proteins in the ER and its swelling, which initiates paraptosis-like cell death (Figure 5).

## Data Availability

The data presented in this study are openly available as Mendeley Data, V1 (doi: 10.17632/fjjtrfv5rv.1 https://data.mendeley.com/datasets/fjjtrfv5rv/1 (accessed on 12 Jul 2022)) and in the Supplementary Material provided here.

## Supplementary Materials

The following supporting information can be downloaded at: https://www.mdpi.com/article/10.3390/membranes12090845/s1, Figure S1: DSFoxy impair the UPS system of HEp-2 cells. (A) Decrease in monoubiquitin level; data from three independent experiments are given. (B) Estimation of 26S proteasome activity of HEp-2 cells during the initiation of their death by 1 mM DDC combined with 25 μM B_12b_. Data from one of five typical experiments are given. (C,D) Bortezomib (BTZ, 1 µM) inhibits this reaction after its addition to 96-plate wells (C) and induces the vacuolization of HEp-2 cells after 24 h of incubation (D); Figure S2: PPIs in protein clusters according to STRING database; Figure S3: PPIs in groups of proteins selected by UniProtKB keywords; Table S1: Total Ub-proteins, data from GO-slim analysis; Table S2: UniProtKB groups of UB-proteins.
