# Supplementary material for: Disulfiram Oxy-Derivatives Suppress Protein Retrotranslocation across the ER Membrane to the Cytosol and Initiate Paraptosis-like Cell Death
Source: Membranes (Basel). 2022 Aug 29;12(9):845. doi: 10.3390/membranes12090845 (PMC9506514; doi:10.3390/membranes12090845)
Supplement: Supplementary file 1 [file membranes-12-00845-s001.zip › membranes-1879652-supplementary figures.pdf]

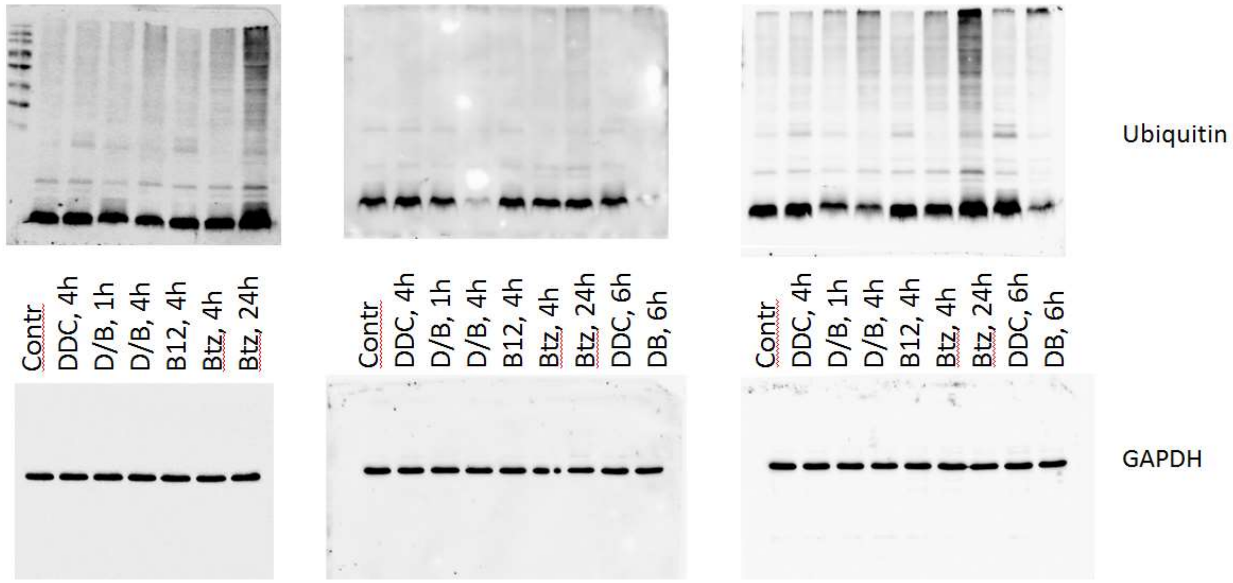
Contr
DDC, 4h
D/B, 1h
D/B, 4h
B12, 4h
Btz, 4h
Btz, 24h
Contr
DDC, 4h
D/B, 1h
D/B, 4h
B12, 4h
Btz, 4h
Btz, 24h
DDC, 6h
DB, 6h
Contr
DDC, 4h
D/B, 1h
D/B, 4h
B12, 4h
Btz, 4h
Btz, 24h
DDC, 6h
DB, 6h
Ubiquitin
GAPDH

A

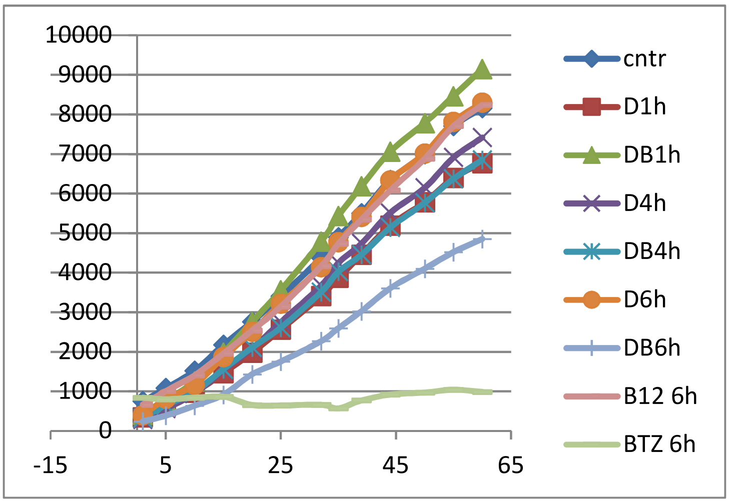
10000
9000
8000
7000
6000
5000
4000
3000
2000
1000
0
-15
5
25
45
65
cntr
D1h
DB1h
D4h
DB4h
D6h
DB6h
B12 6h
BTZ 6h

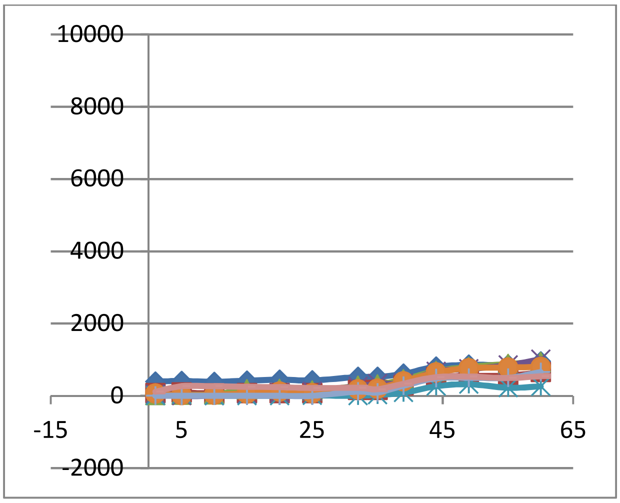
10000
8000
6000
4000
2000
0
-2000
-15
5
25
45
65

B

C

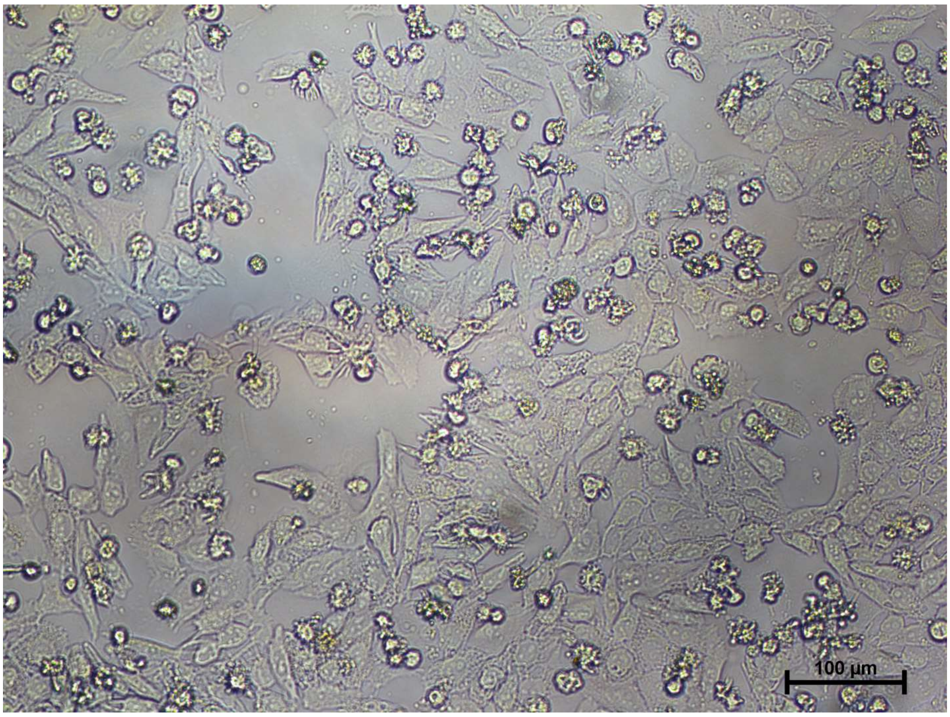

D

Supplementary Figure S1. DSFoxy impaire the UPS system of HEp-2 cells. (A) Decrease in monoubiquitin level, data of 3 independent experiments are given. (B) Estimation of 26S proteasome activity of HEp-2 cells during the initiation of their death by 1 mM DDC combined with 25 µM B_{12b}. Data from one of 5 typical experiments are given. (C,D) Bortezomib (BTZ, 1 µM) inhibits this reaction after its addition to the wells of 96-plate (C) and induces the vacuolization of HEp-2 cells after the 24 h of incubation (D).

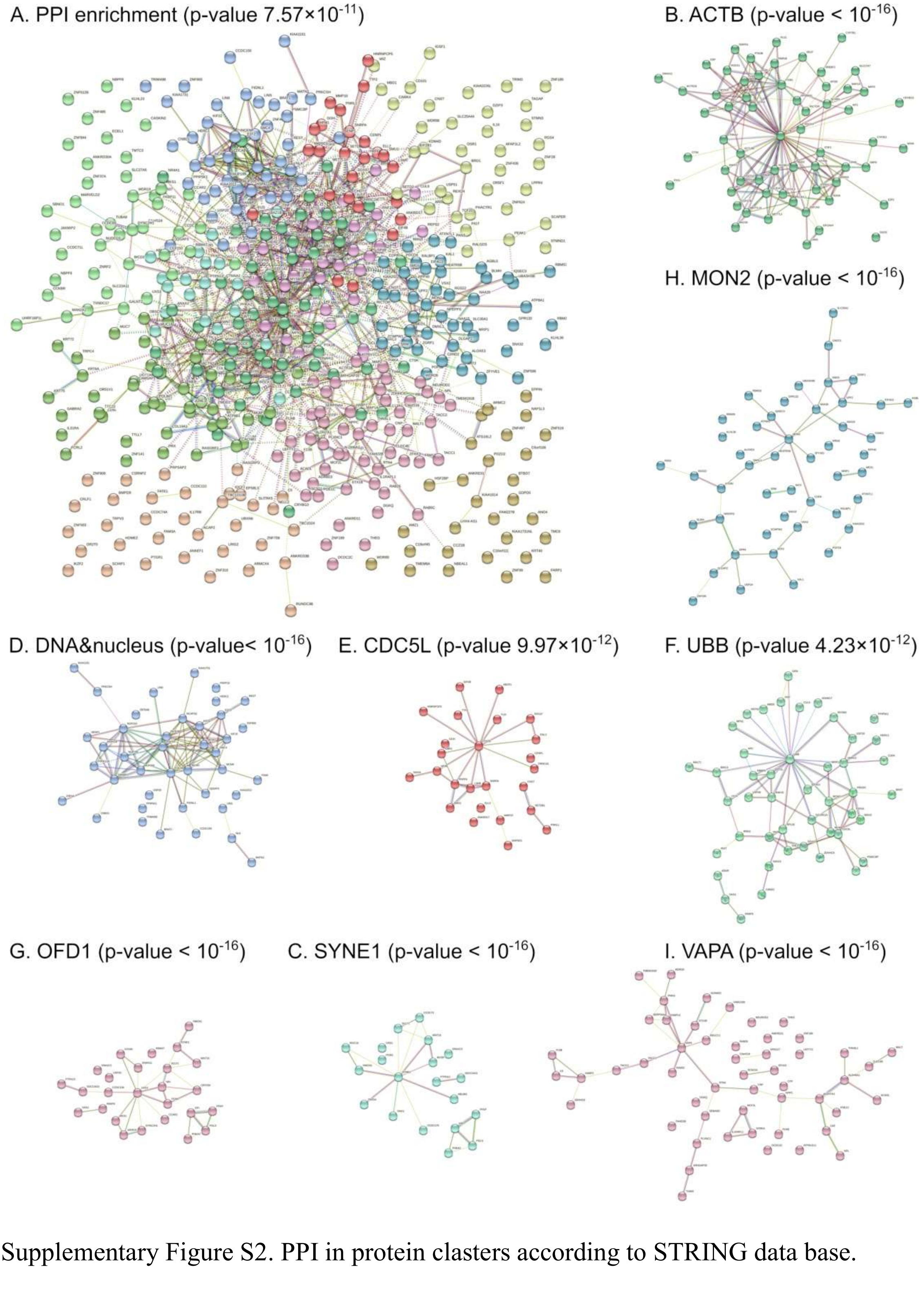

Supplementary Figure S2. PPI in protein clasters according to STRING data base.

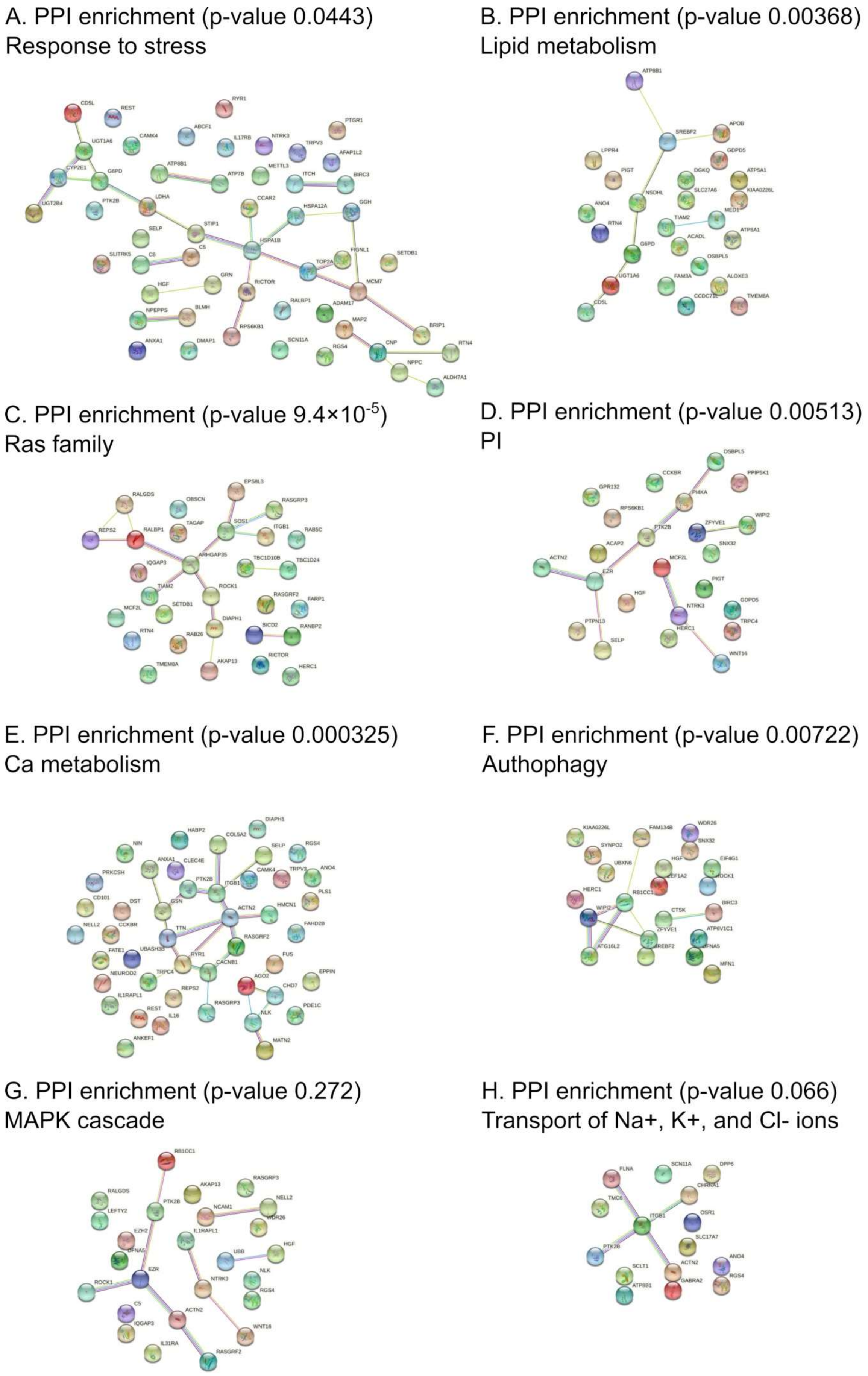

Supplementary Figure S3. PPI in groups of proteins selected by UniProtKB keywords.
